# Association between a regional reimbursement policy and treatment escalation in pulmonary arterial hypertension: a real-world cohort study with long-term Markov cost-effectiveness analysis

**DOI:** 10.3389/fphar.2026.1892453

**Published:** 2026-08-27

**Authors:** Yilin Xie, Yusi Chen, Jun Luo, Jingyuan Chen, Yingjie Tan, Tianyu Wang, Wenjie Chen, Jiang Li

**Affiliations:** Department of Cardiovascular Medicine, The Second Xiangya Hospital of Central South University, Changsha, China

**Keywords:** cost-effectiveness analysis, pharmacoeconomics, pulmonary arterial hypertension, real-world data, reimbursement policy, treatment escalation

## Abstract

**Background:**

Pulmonary arterial hypertension (PAH) requires long-term treatment with high-cost targeted therapies. However, evidence regarding the associations of reimbursement policies with real-world treatment escalation and long-term economic outcomes remains limited.

**Objectives:**

To examine the association between implementation of a regional reimbursement policy and real-world treatment patterns and to evaluate the long-term cost-effectiveness of the observed post-policy treatment distribution compared with the pre-policy distribution.

**Methods:**

A retrospective single-center before-and-after cohort study including 147 patients with PAH was conducted using real-world data from a tertiary hospital in China. Descriptive analyses and multivariable logistic regression were used to examine factors associated with ambrisentan use and combination therapy. A lifetime Markov model incorporating World Health Organization functional class states was developed to compare the observed treatment distributions before and after policy implementation from the Chinese healthcare system perspective. During each model cycle, the time spent in each World Health Organization functional-class state was weighted by the corresponding health utility to calculate quality-adjusted life-years (QALYs).

**Results:**

Following policy implementation, the proportion of patients receiving combination therapy increased from 27.4% to 80.5% (P < 0.001). Policy implementation and ambrisentan use were both associated with receipt of combination therapy, although the policy association was attenuated after adjustment for ambrisentan use. Compared with the pre-policy treatment distribution, the post-policy distribution generated an incremental gain of 0.51 quality-adjusted life years (QALYs) at an additional cost of ¥1,212,269, resulting in an incremental cost-effectiveness ratio (ICER) of ¥2,378,493/QALY. Sensitivity analyses demonstrated that the results were primarily influenced by mortality and utility parameters associated with World Health Organization functional class IV (WHO FC IV). Cost-effectiveness was achieved only when targeted therapy prices were reduced by approximately 90%.

**Conclusion:**

Implementation of the reimbursement policy was associated with substantial changes in real-world treatment patterns among patients with PAH, including increased use of combination therapy. However, under current drug pricing conditions, the post-policy treatment distribution was not cost-effective from the Chinese healthcare system perspective. Substantial reductions in targeted therapy prices may be necessary to improve the economic feasibility of intensified combination treatment strategies.

## 1 Introduction

Pulmonary arterial hypertension (PAH) is a progressive and life-threatening disease characterized by pulmonary vascular remodeling, elevated pulmonary vascular resistance, and right heart failure (Mocumbi et al., 2024). Internationally, Orphanet classifies PAH as a group of rare pulmonary hypertension disorders encompassing idiopathic, heritable, drug- or toxin-induced, and disease-associated forms (Orphanet, n.d.). In China, idiopathic PAH was included in the First Rare Disease List published in 2018 (National Health Commission of the People’s Republic of China, 2018). PAH can occur across a broad age range, including younger and working-age adults. Its early symptoms are often nonspecific and commonly include exertional dyspnea, fatigue, chest discomfort, dizziness, and syncope. As the disease progresses, patients may develop marked exercise limitation, peripheral edema, recurrent hospitalization, and advanced right-heart failure (Farber et al., 2015; Humbert et al., 2022; Mocumbi et al., 2024). Despite substantial advances in diagnostic and therapeutic approaches in recent years, the long-term prognosis of patients with PAH remains unsatisfactory (Farber et al., 2015). With the development of targeted therapies, including endothelin receptor antagonists (ERAs), phosphodiesterase type-5 inhibitors (PDE-5is), and prostacyclin pathway agents, symptom control and long-term outcomes in patients with PAH have improved considerably (Chin et al., 2024). Concurrently, treatment strategies for PAH have progressively shifted from traditional sequential monotherapy toward earlier and more intensive combination therapy. Several randomized controlled trials have demonstrated that initial combination therapy can significantly reduce the risk of disease progression and clinical worsening (Galiè et al., 2015a; Chin et al., 2021). Consequently, the 2022 European Society of Cardiology/European Respiratory Society guidelines on pulmonary hypertension further emphasized a risk-stratification-based early combination therapy strategy (Humbert et al., 2022).

While combination therapy improves clinical outcomes, it is also associated with substantially increased long-term treatment costs. Most patients with PAH require lifelong treatment with one or more high-cost targeted therapies, and the economic burden continues to accumulate as the use of combination therapy increases. In real-world clinical practice, some patients are unable to receive intensified treatment because of financial constraints (Ogbomo et al., 2022; Watzker et al., 2025). Previous studies have shown that PAH-related long-term medication costs and healthcare resource utilization remain substantial, whereas the practical implementation of combination therapy is still limited by factors such as drug accessibility and affordability (Pizzicato et al., 2022; Ramani et al., 2025). Particularly in developing countries with relatively limited healthcare resources, improving access to innovative therapies while maintaining the sustainability of medical insurance funds has become an increasingly important issue in PAH health economics research and healthcare policy decision-making (Dong et al., 2025; Zhu et al., 2025).

In 2018, Hunan Province incorporated ambrisentan into the provincial special-drug reimbursement program for eligible patients with PAH (Human Resources and Social Security Department of Hunan Province, 2018). Following policy implementation, the settlement price of ambrisentan decreased from ¥22,829.40 to ¥14,535.00 per 3-month treatment cycle. Within the principal reimbursement band, 70% of eligible expenses were reimbursed for patients covered by urban employee insurance and 60% for those covered by urban–rural resident insurance. The combined effects of the lower settlement price and reimbursement substantially reduced patients’ out-of-pocket burden and may have made initiation of ambrisentan or its addition to combination therapy more financially feasible. Because this policy preceded the nationwide expansion of reimbursement coverage for PAH-targeted therapies, it provided a relatively independent real-world setting for evaluating the association between reimbursement interventions and treatment pattern changes (National Healthcare Security Administration, 2020).

Greater use of ambrisentan in the present study should not necessarily be interpreted as a one-for-one switch from another targeted therapy. It could represent initiation of targeted therapy, replacement of another agent, or addition of ambrisentan to an existing regimen. For patients, reimbursed access to ambrisentan may reduce direct financial burden and improve practical access to an oral endothelin receptor antagonist, including as part of a guideline-recommended combination regimen (Galiè et al., 2015a; Human Resources and Social Security Department of Hunan Province, 2018; Humbert et al., 2022). For the healthcare system, broader access may improve treatment availability and financial protection (Human Resources and Social Security Department of Hunan Province, 2018; Zhu et al., 2025) and may reduce downstream resource use associated with clinical worsening (Pizzicato et al., 2022; Ramani et al., 2025). However, because PAH treatment is generally long term and combination therapy requires the concurrent use of multiple targeted agents, greater treatment uptake may also substantially increase pharmaceutical expenditure (Ogbomo et al., 2022; Pizzicato et al., 2022; Ramani et al., 2025; Watzker et al., 2025). This trade-off between improved treatment access and increased long-term healthcare costs provided the rationale for the present economic evaluation.

Previous pharmacoeconomic studies of PAH have primarily focused on cost-effectiveness evaluations of single drugs or fixed treatment regimens, with most models constructed using data from randomized controlled trials (Chen et al., 2009; Coyle et al., 2016; Tran-Duy et al., 2021; Buendía et al., 2023; Papademetriou et al., 2023; Dong et al., 2025). Although these studies are valuable for assessing the economic value of specific treatment strategies, they are generally based on predefined treatment regimens or fixed treatment distributions and may therefore fail to adequately capture reimbursement-policy-associated changes in treatment escalation and the use of combination therapy in real-world settings. In practice, policy implementation may be associated not only with the use of a specific core drug but also with differences in combination-therapy use, overall treatment intensity, and the long-term structure of healthcare resource utilization. At present, evidence regarding the long-term economic consequences of reimbursement-policy-associated changes in real-world treatment patterns remains limited, particularly in China. In particular, no previous study has specifically evaluated the long-term economic consequences of the observed redistribution of treatment intensity among supportive treatment, monotherapy, dual therapy, and triple therapy before and after reimbursement policy implementation.

Accordingly, using a real-world cohort of patients with PAH in Hunan Province, we combined descriptive analyses and multivariable logistic regression with a Markov decision model. We examined the association between implementation of the regional reimbursement policy and real-world treatment patterns and subsequently estimated the projected long-term costs, QALYs, and cost-effectiveness of the observed post-policy treatment distribution compared with the pre-policy distribution. Unlike previous cost-effectiveness studies primarily focused on individual drugs, the present study examined the associations among reimbursement policy implementation, real-world treatment patterns, and projected long-term economic outcomes. The findings of this study may provide real-world evidence for optimizing reimbursement policies for high-cost PAH therapies and may help inform future reimbursement policy optimization for high-cost therapies in rare diseases.

## 2 Methods

### 2.1 Study design and data source

This study employed a retrospective real-world design combined with a decision-analytic model to evaluate changes in treatment patterns and long-term economic outcomes among patients with PAH before and after the implementation of the reimbursement policy. Real-world data were obtained from the electronic medical record database of The Second Xiangya Hospital of Central South University, a tertiary referral hospital with a dedicated pulmonary vascular disease subspecialty and multidisciplinary team providing standardized evaluation and management for patients with PAH. A total of 147 patients who met the diagnostic criteria for PAH according to the 2015 ESC/ERS guidelines applicable during the study period were included (Galiè et al., 2015b). Detailed diagnostic criteria and the corresponding reference are provided in Supplementary Table S1.

The study period and policy groups were defined as follows. Ambrisentan was formally included in the Hunan provincial special-drug reimbursement program on 1 August 2018. Eligible patients were identified between 1 January 2016, and 1 August 2019. The final cohort therefore represented patients identified over a 43-month period at a single institution. The index date was defined as the date of the first eligible hospitalization, during which baseline clinical characteristics and the PAH-targeted treatment regimen were ascertained. Patients with an index date between 1 January 2016, and 31 July 2018, were classified into the pre-policy group, whereas those with an index date on or after 1 August 2018, were classified into the post-policy group. Each patient contributed only one index record. The treatment regimen was defined according to the PAH-targeted therapy prescribed at discharge from the index hospitalization. For patients whose subsequent follow-up extended beyond the policy implementation date, group assignment remained based on the index date, and subsequent treatment changes were not used to redefine policy exposure or the index treatment regimen. No formal transition period was applied in the primary analysis. A pharmacological washout period was not applicable because the exposure of interest was implementation of a reimbursement policy rather than initiation or discontinuation of a medication.

Demographic characteristics, World Health Organization (WHO) functional class (FC), medication use, and insurance status were collected. This study was approved by the Ethics Committee of the Second Xiangya Hospital of Central South University (Approval No. LYF20250180). The requirement for informed consent was waived because of the retrospective nature of the study.

### 2.2 Statistical analysis

Descriptive analyses were first conducted to compare baseline characteristics and treatment patterns before and after implementation of the policy. Continuous variables were presented as means ± standard deviations, while categorical variables were expressed as frequencies and percentages. Between-group comparisons were performed using the independent-samples t-test, chi-square test, or Fisher’s exact test, as appropriate.

Subsequently, univariable analyses were conducted using ambrisentan utilization and treatment regimen distribution (no targeted therapy, monotherapy, dual combination therapy, and triple combination therapy) as outcome variables to evaluate their associations with clinical and demographic characteristics. Variables with a P value <0.20 in the univariable analyses were included in subsequent multivariable analyses.

Based on these results, multivariable binary logistic regression models were constructed. In addition to variables identified as potentially relevant in the univariable analyses, prespecified clinically relevant confounders, including insurance type and baseline FC classification, were also incorporated into the models for adjustment. All models were fitted using the enter method.

Three multivariable binary logistic regression models were fitted for two outcomes. In Model 1, ambrisentan use (yes = 1, no = 0) was used as the dependent variable to examine its association with reimbursement policy implementation. For the treatment-escalation analysis, treatment patterns were dichotomized into combination therapy (dual or triple therapy) versus non-combination therapy (monotherapy or no targeted therapy), because the limited sample sizes in some treatment categories could have resulted in unstable estimates in a multinomial model.

Our hypothesized conceptual framework was that reimbursement policy implementation could be associated with combination therapy partly through increased use of ambrisentan and partly through other contemporaneous changes in treatment access and clinical practice. Two sequential multivariable binary logistic regression models were therefore fitted for the combination-therapy outcome. Model 2A included age group, baseline WHO functional class, comorbidities, occupation, insurance type, and reimbursement policy implementation, without adjustment for ambrisentan use. Model 2B additionally included ambrisentan use as a secondary exploratory model. Because ambrisentan use and treatment regimen were ascertained during the same index hospitalization, and ambrisentan itself was a component of the treatment regimen, comparison of the two models was not interpreted as identifying direct or indirect causal effects or as a formal mediation analysis.

Regression results were reported as odds ratios (ORs) with corresponding 95% confidence intervals (CIs). All statistical tests were two-sided, and P < 0.05 was considered statistically significant. All analyses were performed using IBM SPSS Statistics software (version 26). Given the sparse-event distribution in several subgroups, regression results were interpreted cautiously with emphasis on association direction rather than precise effect-size estimation.

### 2.3 Model structure

**FIGURE 1:**
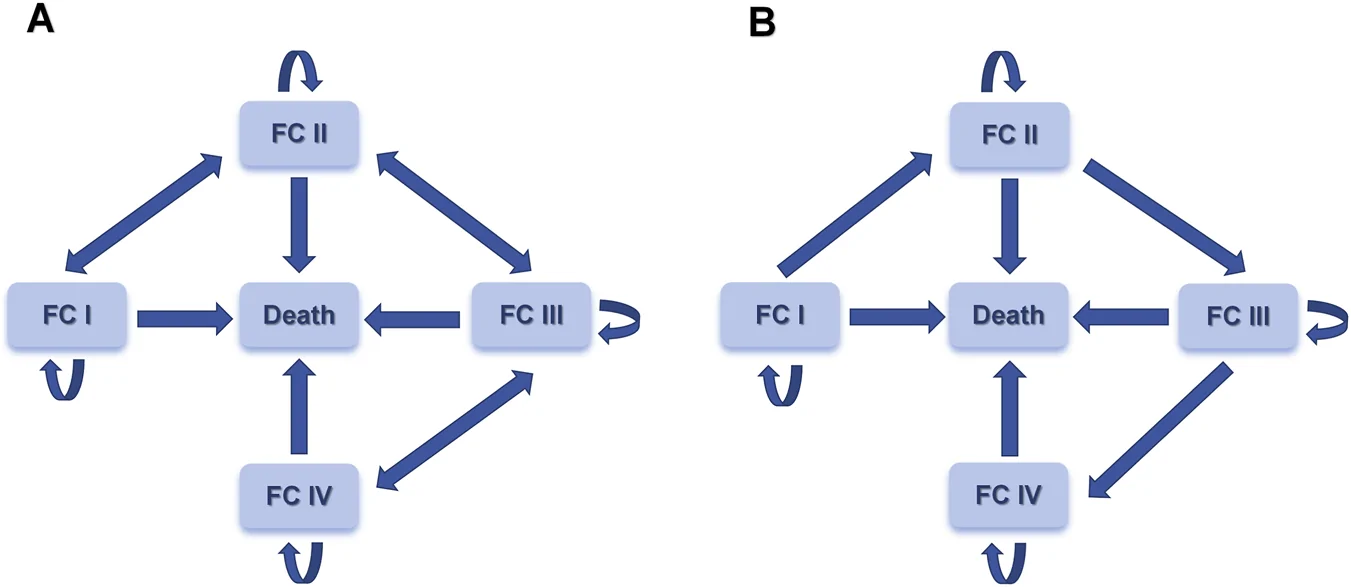
Markov models of the **(A)** first and **(B)** subsequent cycles. Each rectangle represents a state, lines and arrows represent transition directions between states. Patients can be in only one health state per cycle. FC, functional class.

From the perspective of the Chinese healthcare system, a Markov model was constructed using TreeAge Pro to simulate long-term disease progression and economic outcomes in patients with PAH. The WHO functional classification, adapted from the New York Heart Association functional classification, was used to reflect disease severity in PAH. Based on disease progression, the model consisted of five mutually exclusive Markov health states: WHO FC I, II, III, IV, and death (absorbing state) (Chen et al., 2009; Tran et al., 2015; Coyle et al., 2016). Considering the progressive nature of PAH, patients were allowed to transition to improved, stable, or worsened states during the first cycle. In subsequent cycles, patients could only remain in the current state, deteriorate to the next worse adjacent state, or transition to death (Chen et al., 2009; Tran et al., 2015; Dong et al., 2023). The initial state distribution was derived from the real-world cohort included in this study. A lifetime horizon was adopted to fully capture long-term disease progression and treatment benefits, with a cycle length of 3 months (Dong et al., 2025) (Figure 1).

Unlike conventional pharmacoeconomic studies comparing fixed treatment regimens, the present model was designed to compare two real-world treatment distribution scenarios derived from observed clinical practice before and after reimbursement policy implementation. Therefore, the model structure explicitly incorporated differences in treatment intensity distributions across supportive treatment, monotherapy, dual therapy, and triple therapy branches. Dual and triple therapy were retained as separate treatment branches in the Markov model and were combined only for the binary logistic regression analysis.

### 2.4 Clinical inputs

A targeted literature review was conducted to identify appropriate model input parameters, as summarized in Table 1. Transition probabilities between health states were derived from previously published clinical and health economic studies. As long-term transition probability data covering WHO FC I–IV patients with PAH who had not received targeted therapy are currently lacking in China, the transition probabilities for supportive treatment were extracted from a previously published model-based economic evaluation conducted in an Asian setting (Lilyasari et al., 2019). The source study reported identical transition probabilities for FC I to FC II and FC II to FC III, and identical probabilities for FC II to FC I and FC III to FC II; these values were adopted directly rather than independently assumed or estimated in the present study.

**TABLE 1:** Basic parameters input to the model and the ranges of the sensitivity analyses.

Parameter	Base-case value	Lower limit	Upper limit	Distribution	Source
Treatment efficacy & transition probabilities (per 3-month cycle)					
Supportive treatment					
FC I → FC II transition probability	0.127	0.095	0.159	Beta	Lilyasari et al. (2019)
FC II → FC I transition probability	0.125	0.094	0.156	Beta	Lilyasari et al. (2019)
FC II → FC III transition probability	0.127	0.095	0.159	Beta	Lilyasari et al. (2019)
FC III → FC II transition probability	0.125	0.094	0.156	Beta	Lilyasari et al. (2019)
FC III → FC IV transition probability	0.094	0.071	0.118	Beta	Lilyasari et al. (2019)
FC IV → FC III transition probability	0.025	0.019	0.031	Beta	Lilyasari et al. (2019)
Monotherapy					
Weighted relative risk (RR) for FC improvement	2.8157	2.1118	3.5196	Log-normal	Coyle et al. (2016)
Weighted relative risk (RR) for FC worsening	0.3851	0.2888	0.4814	Log-normal	Coyle et al. (2016)
Dual therapy					
Hazard ratio (HR) for disease worsening	0.500	0.350	0.720	Log-normal	Galiè et al. (2015a)
Triple therapy					
Hazard Ratio (HR) for disease worsening	0.590	0.320	1.090	Log-normal	Chin et al. (2021)
3-month mortality probabilities by functional class					
Mortality probability for FC I	0.000729	0.000547	0.000911	Beta	Thenappan et al. (2010), Office of the Leading Group of the State Council for the Seventh National Population Census (2022)
Mortality probability for FC II	0.003142	0.000965	0.010459	Beta	Thenappan et al. (2010), Office of the Leading Group of the State Council for the Seventh National Population Census (2022)
Mortality probability for FC III	0.005524	0.001779	0.017576	Beta	Thenappan et al. (2010), Office of the Leading Group of the State Council for the Seventh National Population Census (2022)
Mortality probability for FC IV	0.008056	0.002570	0.025650	Beta	Thenappan et al. (2010), Office of the Leading Group of the State Council for the Seventh National Population Census (2022)
Health utilities					
Utility of FC I	0.730	0.640	0.820	Beta	Keogh et al. (2007)
Utility of FC II	0.670	0.570	0.770	Beta	Keogh et al. (2007)
Utility of FC III	0.600	0.500	0.700	Beta	Keogh et al. (2007)
Utility of FC IV	0.520	0.430	0.610	Beta	Keogh et al. (2007)
Medical costs per 3-month cycle (¥)					
Outpatient registration cost	57.45	43.09	71.81	Gamma	Dong et al. (2023)
Follow-up examination cost	620.91	465.68	776.14	Gamma	Dong et al. (2023)
Hospitalization cost (FC I-III)	4,393.73	3,295.30	5,492.16	Gamma	Dong et al. (2023)
Hospitalization cost (FC IV)	8,787.48	6,590.61	10,984.35	Gamma	Dong et al. (2023)
Drug costs per 3-month cycle (¥)					
Ambrisentan (pre-policy)	22,829.40	17,122.05	28,536.75	Gamma	YaoZh (2026)
Ambrisentan (post-policy)	14,535.00	10,901.25	18,168.75	Gamma	Human Resources and Social Security Department of Hunan Province (2018)
Bosentan	12,603.60	9,452.70	15,754.50	Gamma	YaoZh (2026)
Sildenafil	4,437.18	3,327.89	5,546.48	Gamma	YaoZh (2026)
Tadalafil	18,002.70	13,502.03	22,503.38	Gamma	YaoZh (2026)
Macitentan	89,940.00	67,455.00	112,425.00	Gamma	YaoZh (2026)
Treprostinil	52,524.00	39,393.00	65,655.00	Gamma	YaoZh (2026)
Other parameters					
Discount rate	5.0%	0.0%	8.0%	—	Liu et al. (2020)

All costs are expressed in 2018 Chinese yuan per 3-month cycle.

Drug-specific relative risks (RRs) for World Health Organization functional-class (WHO FC) improvement and worsening under monotherapy were extracted directly from a network meta-analysis (Coyle et al., 2016). The published RRs and corresponding 95% credible intervals were used as reported. No odds ratios or raw event counts were converted into RRs in the present study. Because monotherapy was represented as a single aggregated treatment-intensity category in the Markov model, a composite efficacy estimate was calculated using the observed drug-utilization distribution among all 47 patients receiving monotherapy. For outcome k, the weighted RR was calculated as:

$${\bar{RR}}_{k}=\sum_{i=1}^{m}w_{i}RR_{i,k}=\frac{\sum_{i=1}^{m}n_{i}RR_{i,k}}{\sum_{i=1}^{m}n_{i}},$$

Where *k* denoted WHO FC improvement or worsening, *n*_*i*_ was the number of patients receiving drug *i*, *w*_*i*_ was the corresponding utilization proportion, and *RR*_*i,k*_ was the drug-specific RR for WHO FC improvement or worsening reported in the source study. Because each drug-specific RR was applied to the same baseline transition probability, weighting the RRs was algebraically equivalent to weighting the corresponding drug-specific transition probabilities.

Among the 47 patients receiving monotherapy, 26 received bosentan, one received ambrisentan, 17 received sildenafil, two received tadalafil, and one received subcutaneous treprostinil. Because a corresponding short-term RR was unavailable for subcutaneous treprostinil, the published epoprostenol estimate was used as a prostacyclin-class proxy (Tran et al., 2015; Coyle et al., 2016). Detailed drug-utilization proportions, drug-specific RRs, and numerical calculations are provided in Supplementary Table S4.

The resulting composite RRs were 2.8157 for WHO FC improvement and 0.3851 for WHO FC worsening. A common composite monotherapy RR was applied to both policy scenarios because monotherapy was modeled as the same treatment-intensity category in the two scenarios rather than as separate drug-specific states. Differences in drug composition between the policy scenarios were therefore incorporated into treatment costs rather than into policy-specific monotherapy transition matrices.

In addition, considering the insufficient evidence regarding an independent survival benefit associated with targeted therapy, different treatment patterns were assumed not to directly affect mortality risk but instead improve long-term outcomes indirectly by delaying disease progression. This conservative assumption was adopted to avoid double counting of treatment effects because functional-class-dependent mortality was already incorporated within the model structure (Macchia et al., 2007; Coyle et al., 2016).

Efficacy parameters for combination therapy were obtained directly, as reported, from the original randomized controlled trials and were expressed as hazard ratios (HRs). No odds ratios or raw event data were converted into HRs in the present study. Because high-quality long-term real-world comparative effectiveness data for PAH combination therapy remain limited, the reported HRs were applied to the model using the standard discretization formula:

$$P_{\mathrm{treatment}}={1-\left(1-P_{\mathrm{treatment}}\right)}^{\mathrm{HR}},$$

where *P*_*reference*_ represented the 3-month worsening probability under the reference treatment.

Parameters for dual combination therapy were derived from the AMBITION trial, in which the HR for disease worsening was 0.50 (95% CI, 0.35–0.72) (Galiè et al., 2015a). Because no significant difference in functional improvement was observed, this study conservatively assumed that the probability of improvement under dual therapy was identical to that of monotherapy, while only deterioration probabilities were adjusted. Parameters for triple combination therapy were obtained from the TRITON trial, which reported an HR of 0.59 (95% CI, 0.32–1.09) for disease worsening compared with dual therapy (Chin et al., 2021). Similarly, because of the lack of clear evidence for further functional improvement, the probability of improvement under triple therapy was assumed to be the same as that under dual therapy.

Background mortality rates for different FC states were estimated by combining published mortality risk ratios for each FC state relative to the general population (Thenappan et al., 2010) with life-table data from China’s Seventh National Population Census in 2020 (Office of the Leading Group of the State Council for the Seventh National Population Census, 2022), adjusted according to the age and sex distribution of the initial cohort. The following formula was used to calculate mortality probabilities for each FC state within each cycle: p = 1−e^-rt^ where r represents the rate, p represents the probability, and t represents one 3-month model cycle (i.e., 0.25 years when r is expressed as an annual rate) (Fleurence and Hollenbeak, 2007).

### 2.5 Costs and utilities

From the perspective of the Chinese healthcare system, only direct medical costs were included, including medication, outpatient, follow-up examination, and hospitalization costs. All costs were measured in 2018 Chinese Yuan (CNY) and converted to 3-month cycle costs.

#### 2.5.1 Cost calculation framework

To reflect differences in real-world treatment patterns observed before and after reimbursement policy implementation, cycle costs were estimated using a simplified two-stage approach.

Stage 1: Weighted drug cost estimation. Within each treatment branch (supportive treatment, monotherapy, dual therapy, and triple therapy), drug cycle costs were calculated by weighting the costs of individual regimens according to their observed utilization proportions in the real-world cohort. The formula was as follows:

$$C_{drug,m}=\sum_{i=1}^{n}w_{i}\cdot c_{i}$$

Where $C_{drug,m}$ represents the weighted drug cost for treatment pattern *m*; $w_{i}$ represents the utilization proportion of the *i*th drug or regimen; and $c_{i}$ represents the corresponding cycle cost of the drug or regimen.

Stage 2: Addition of FC-specific non-drug costs. Because the model structure explicitly separated treatment branches, total cycle costs for each health state were calculated by simply adding the FC-specific non-drug medical costs to the corresponding weighted drug costs:

$$C_{s,FC}=C_{drug,s}+C_{med,FC}$$

Where $C_{s,FC}$ represents the total cycle cost of treatment branch *s* in a given FC state; $C_{drug,s}$ represents the weighted drug cost associated with the treatment branch; and $C_{med,FC}$ represents the non-drug medical cost associated with the corresponding FC state.

#### 2.5.2 Drug costs

Drug costs were primarily derived from local reimbursement standards and centralized drug procurement price data from Hunan Province (Human Resources and Social Security Department of Hunan Province, 2018; YaoZh, 2026). Separate pre- and post-policy settlement prices were applied for the core intervention drug ambrisentan. Because the base-case analysis was conducted from the Chinese healthcare-system perspective, the full post-policy settlement price of ¥14,535 per 3-month cycle was included, irrespective of how this cost was divided between the insurance fund and the patient. For drugs lacking local pricing data, provincial bid-winning prices from the same period were used as proxy estimates. All medication cycle costs were calculated based on the defined daily dose (DDD) recommended in PAH clinical guidelines and prescribing information (Humbert et al., 2022). Drug acquisition costs were based on reimbursement or procurement prices applicable in 2018 and therefore required no further inflation adjustment.

For the high-cost targeted therapy treprostinil, because dosing requires continuous titration according to body weight and tolerability, resulting in substantial interpatient variability, a micro-costing approach was adopted to more accurately reflect the actual economic burden in real-world practice (Liu et al., 2020).

To ensure model rigor, targeted drug costs in the supportive treatment branch were set to zero, thereby allowing estimation of the true incremental costs of targeted therapy relative to supportive treatment.

#### 2.5.3 Other non-drug medical costs

According to routine PAH management practices in China, patients undergo regular outpatient follow-up and clinical evaluations. For routine outpatient and examination costs that were incompletely documented in real-world medical records, localized standard cost estimates based on publicly available reimbursement catalogues from medical insurance bureaus in cities including Changsha were adopted (Dong et al., 2023).

Hospitalization costs were stratified according to FC status. Considering that hospitalization expenses recorded in the hospital information system might include targeted therapy costs, hospitalization cost data excluding drug expenditures were obtained from published literature to avoid double counting (Dong et al., 2023).

For consistency with the common price year, the non-drug medical cost estimates obtained from Dong et al. were adjusted to 2018 values using the healthcare component of the Chinese Consumer Price Index (CPI). A cumulative deflation factor of 0.949775 was applied (National Bureau of Statistics of China, n.d.).

#### 2.5.4 Utilities

Utility values corresponding to different WHO FC states were obtained from a study that mapped SF-36 scores to utility estimates, and were used to calculate quality-adjusted life years (QALYs) (Keogh et al., 2007). In the absence of Chinese population-specific utility estimates for PAH, internationally validated utility values were adopted, which is consistent with previous Chinese pharmacoeconomic studies in PAH, and their uncertainty was further assessed in sensitivity analyses.

### 2.6 Cost-effectiveness analysis

Model outputs included total costs, QALYs, and incremental cost-effectiveness ratios (ICERs). Both costs and health outcomes were discounted at an annual rate of 5%, while discount rates of 0% and 8% were examined in sensitivity analyses (Liu et al., 2020). The willingness-to-pay (WTP) threshold was set at three times the *per capita* gross domestic product of China in 2018 (¥193,932/QALY) (National Bureau of Statistics of China, 2019; Liu et al., 2020). The 2018 GDP value was selected to align the WTP threshold with both the year of policy implementation and the common price year used for the cost inputs. The China Guidelines for Pharmacoeconomic Evaluations recommend a threshold range of one to three times national *per capita* GDP per QALY. The upper bound of this range was used because it provides a more favorable benchmark for the post-policy strategy and therefore represents a conservative basis for assessing its cost-effectiveness. An ICER below this threshold was considered cost-effective.

### 2.7 Sensitivity analyses

To assess the robustness of the model results, both one-way sensitivity analysis and probabilistic sensitivity analysis were conducted. In the one-way sensitivity analysis, parameters were varied across their corresponding 95% confidence or credible intervals, with upper and lower bounds derived from the literature (Liu et al., 2020). If parameter ranges were unavailable, a ±25% variation around the base-case value was assumed (Dong et al., 2023). In the base-case analysis, the observed monotherapy utilization proportions were treated as fixed cohort-derived weighting inputs because they represented the actual treatment composition observed in the study cohort. The drug-specific RRs were obtained from the same network meta-analysis and may therefore be statistically correlated. However, the full variance–covariance structure required to propagate these correlations into the weighted composite estimate was unavailable. Independently sampling the individual RRs without accounting for their correlation could misrepresent the uncertainty of the composite parameter. Therefore, the individual RRs and utilization proportions were not sampled separately, and residual uncertainty was represented at the level of the derived composite RRs using a ±25% range. Results were presented as tornado diagrams.

Probabilistic sensitivity analysis was conducted using Monte Carlo simulation with 5,000 iterations. Transition probabilities and utility values were assigned beta distributions, cost parameters were assigned gamma distributions, and RRs and HRs were assigned log-normal distributions (Table 1) (Briggs et al., 2012). Results were presented as scatter plots on the cost-effectiveness plane and cost-effectiveness acceptability curves (CEACs).

### 2.8 Scenario analysis

To evaluate the effects of key modeling assumptions and parameter variations on study outcomes, several prespecified scenario analyses were conducted.

First, drug price scenario analyses were conducted by systematically adjusting the prices of targeted therapies. These included extreme price-reduction scenarios for individual high-cost drugs (treprostinil and ambrisentan), hypothetical cost-input scenarios in which the post-policy ambrisentan cost was reduced to 30% or 40% of its base-case settlement price, and scenarios in which the prices of all targeted therapies were uniformly reduced by 50%, 70%, 80%, and 90%. The 30% and 40% values corresponded to the estimated patient cost-sharing proportions under urban employee insurance and urban–rural resident insurance, respectively. These were exploratory cost-input scenarios rather than complete analyses from an alternative costing perspective.

On this basis, threshold analyses were further conducted. A one-way threshold analysis was performed with the overall price of targeted therapies treated as a continuous variable to identify the minimum price reduction required for the ICER to fall below the WTP threshold.

In addition, to examine the model’s dependence on long-term assumptions, alternative time horizons of 5 and 10 years were evaluated and compared with the lifetime horizon results.

Finally, disease-stage scenario analyses were constructed by modifying the initial state distribution of the model, including an early-intervention scenario in which all patients entered the model in FC II at baseline and a late-intervention scenario in which all patients entered the model in FC III at baseline, in order to evaluate the impact of treatment timing on economic outcomes.

### 2.9 Model validation

Supportive external validation was conducted by comparing the model-predicted 1-, 3-, and 5-year cumulative mortality risks with observed outcomes from a national multicenter prospective registry of Chinese patients with pulmonary arterial hypertension associated with congenital heart disease. The external registry included 1,060 patients and reported 1-, 3-, and 5-year survival rates of 96.9%, 92.9%, and 87.6%, respectively, corresponding to cumulative mortality risks of 3.1%, 7.1%, and 12.4% (Xie et al., 2023). Model-predicted mortality risks were extracted from the Markov cohort traces at cycles 4, 12, and 20, corresponding to 1, 3, and 5 years. Because the external registry specifically included patients with PAH associated with congenital heart disease, the comparison was considered supportive external validation rather than formal patient-level validation.

## 3 Results

### 3.1 Baseline characteristics

A total of 147 patients with PAH were included in this study, of whom 67.3% were female, with a mean age of 35.4 ± 17.3 years. No statistically significant differences were observed between the pre-policy and post-policy groups in key baseline characteristics, including age, sex, and baseline functional class (all P > 0.05). However, the distribution of insurance types differed significantly between the two groups (P < 0.001). Baseline demographic and disease severity characteristics were generally comparable between groups, supporting the validity of subsequent treatment-pattern comparisons (Table 2).

**TABLE 2:** Baseline demographic and clinical characteristics of the study population before and after policy implementation.

Variable	Overall (n = 147)	Pre-policy (n = 106)	Post-policy (n = 41)	P value
Demographic characteristics				
Age, year	35.4 ± 17.3	34.1 ± 17.0	38.9 ± 17.8	0.129
Female, n (%)	99 (67.3)	72 (67.9)	27 (65.9)	0.810
Clinical characteristics				
WHO FC, n (%)				
I	2 (1.4)	2 (1.9)	0 (0.0)	0.585
II	45 (30.6)	35 (33.0)	10 (24.4)	—
III	74 (50.3)	52 (49.1)	22 (53.7)	—
IV	26 (17.7)	17 (16.0)	9 (22.0)	—
NT-proBNP, n (%)				
<300 ng/mL	8 (5.4)	5 (4.7)	3 (7.3)	0.742
300–1,100 ng/mL	26 (17.7)	20 (18.9)	6 (14.6)	—
>1,100 ng/mL	113 (76.9)	81 (76.4)	32 (78.0)	—
Comorbidities, n (%)				
0	64 (43.5)	49 (46.2)	15 (36.6)	0.549
1–2	56 (38.1)	39 (36.8)	17 (41.5)	—
≥3	27 (18.4)	18 (17.0)	9 (22.0)	—
Treatment characteristics				
Treatment regimens, n (%)				
No targeted therapy	38 (25.9)	37 (34.9)	1 (2.4)	<0.001
Monotherapy	47 (32.0)	40 (37.7)	7 (17.1)	—
Dual therapy	52 (35.4)	28 (26.4)	24 (58.5)	—
Triple therapy	10 (6.8)	1 (0.9)	9 (22.0)	—
Combination therapy, n (%)	62 (42.2)	29 (27.4)	33 (80.5)	<0.001
Ambrisentan use, n (%)	26 (17.7)	2 (1.9)	24 (58.5)	<0.001
Bosentan use, n (%)	59 (40.1)	51 (48.1)	8 (19.5)	0.002
Other characteristics				
Type of insurance, n (%)				
Urban employee	15 (10.2)	7 (6.6)	8 (19.5)	<0.001
Urban-rural resident	52 (35.4)	31 (29.2)	21 (51.2)	—
Out-of-pocket	80 (54.4)	68 (64.2)	12 (29.3)	—
Location of residence, n (%)				
Urban	53 (36.1)	34 (32.1)	19 (46.3)	0.106
Rural	94 (63.9)	72 (67.9)	22 (53.7)	—
Occupation, n (%)				
Employed	29 (19.7)	19 (17.9)	10 (24.4)	0.547
Retired	9 (6.1)	6 (5.7)	3 (7.3)	—
Unemployed/Farmer	109 (74.1)	81 (76.4)	28 (68.3)	—

Abbreviations: WHO FC, World Health Organization Functional Class; NT-proBNP, N-terminal pro-brain natriuretic peptide.

### 3.2 Association between reimbursement policy implementation and treatment patterns

Following implementation of the reimbursement policy, treatment patterns involving targeted therapies among patients with PAH changed substantially. The proportion of patients receiving combination therapy increased significantly from 27.4% before policy implementation to 80.5% after implementation (P < 0.001), among which the proportion receiving triple combination therapy markedly increased from 0.9% to 22.0%. Meanwhile, the proportion of patients receiving dual combination therapy also increased substantially, whereas the proportions of patients receiving no targeted therapy or monotherapy decreased significantly. Overall, real-world treatment patterns were observed to shift from lower-intensity therapy toward more intensive combination therapy following policy implementation (Table 2).

### 3.3 Factors associated with treatment escalation

To further explore the potential pathways underlying the observed associations between reimbursement policy implementation and treatment patterns, univariable analyses were first performed (see Supplementary Tables S2, S3). The results demonstrated that variables including reimbursement policy and insurance type were significantly associated with both ambrisentan use and targeted treatment regimens (P < 0.05). Subsequently, multivariable logistic regression models were constructed.

In Model 1, after adjustment for the included covariates, reimbursement policy implementation remained the factor most strongly associated with ambrisentan use (OR = 179.818, 95% CI: 19.196–1,684.483, P < 0.001) (Table 3). For receipt of combination therapy, Model 2A did not include ambrisentan use. In this model, reimbursement policy implementation was associated with receipt of combination therapy (OR = 11.068, 95% CI: 3.863–31.715, P < 0.001). After ambrisentan use was added in Model 2B, the association between policy implementation and combination therapy was attenuated but remained statistically significant (OR = 4.505, 95% CI: 1.324–15.325, P = 0.016). Ambrisentan use was also associated with receipt of combination therapy (OR = 19.903, 95% CI: 1.696–233.623, P = 0.017) (Table 4).

**TABLE 3:** Multivariable binary logistic regression analysis of factors associated with ambrisentan use.

Variable	Category	P value	OR	95% CI	
WHO FC	III	0.046	6.741	1.035	43.922
	IV	0.090	7.595	0.728	79.242
	I–II	—	Ref	—	—
Location of residence	Rural	0.530	0.555	0.088	3.488
	Urban	—	Ref	—	—
Occupation	Employed	0.006	53.510	3.205	893.358
	Retired	0.056	25.640	0.917	716.639
	Unemployed/Farmer	—	Ref	—	—
Type of insurance system	Urban employee	0.291	0.244	0.018	3.347
	Urban-rural resident	0.002	26.379	3.182	218.687
	Out-of-pocket	—	Ref	—	—
Implementation of the policy	After	<0.001	179.818	19.196	1,684.483
	Before	—	Ref	—	—

Wide confidence intervals may reflect sparse-event distribution in certain subgroups.

**TABLE 4:** Sequential multivariable binary logistic regression analyses of factors associated with combination therapy.

Patient characteristics	Category	Model 2A OR (95% CI)	P value	Model 2B OR (95% CI)	P value
Age, years	<26	1.407 (0.325–6.091)	0.648	2.590 (0.432–15.522)	0.298
	26–34	2.890 (0.640–13.051)	0.168	5.699 (0.892–36.430)	0.066
	35–46	1.730 (0.418–7.161)	0.449	3.740 (0.639–21.890)	0.143
	>46	Ref	—	Ref	—
WHO FC	III	5.630 (1.926–16.457)	0.002	4.870 (1.616–14.674)	0.005
	IV	4.010 (1.028–15.644)	0.046	3.312 (0.791–13.873)	0.101
	I–II	Ref	—	Ref	—
Comorbidities	0	0.892 (0.221–3.606)	0.873	0.749 (0.170–3.307)	0.703
	1–2	0.460 (0.127–1.661)	0.236	0.443 (0.111–1.762)	0.248
	≥3	Ref	—	Ref	—
Occupation	Employed	2.828 (0.861–9.282)	0.087	1.811 (0.505–6.489)	0.362
	Retired	3.650 (0.436–30.519)	0.232	4.797 (0.460–50.066)	0.190
	Unemployed/Farmer	Ref	—	Ref	—
Type of insurance system	Urban employee	3.301 (0.554–19.667)	0.190	4.096 (0.610–27.497)	0.147
	Urban-rural resident	3.566 (1.377–9.234)	0.009	2.724 (1.022–7.258)	0.045
	Out-of-pocket	Ref	—	Ref	—
Implementation of the policy	After	11.068 (3.863–31.715)	<0.001	4.505 (1.324–15.325)	0.016
	Before	Ref	—	Ref	—
Ambrisentan use	Used	Not included	—	19.903 (1.696–233.623)	0.017
	Unused	—	—	Ref	—

Model 2A included age group, baseline WHO FC, comorbidities, occupation, insurance type, and reimbursement policy implementation, without adjustment for ambrisentan use. Model 2B additionally included ambrisentan use. The comparison between the two models was exploratory and was not interpreted as a formal mediation analysis. Wide confidence intervals may reflect sparse-event distributions in certain subgroups.

The change in the policy estimate was descriptively consistent with the hypothesized pathway involving ambrisentan use; however, it was not interpreted as evidence of formal mediation.

### 3.4 Cost-effectiveness analysis

Over the lifetime simulation horizon, although the post-policy treatment distribution scenario generated measurable health gains compared with the pre-policy scenario, these benefits were accompanied by substantial increases in long-term healthcare expenditures. The post-policy treatment distribution scenario resulted in an incremental cost of approximately ¥1,212,268.95 and an incremental gain of 0.51 QALYs, corresponding to an ICER of ¥2,378,492.79/QALY. This ICER substantially exceeded the predefined WTP threshold, defined as three times the annual *per capita* gross domestic product of China (¥193,932/QALY), indicating that the policy-associated treatment pattern scenario was not cost-effective under the prevailing drug pricing structure (Table 5).

**TABLE 5:** Base-case cost-effectiveness analysis results comparing pre-policy and post-policy treatment distribution scenarios.

Treatment distribution scenario	Cost (¥)	Incremental cost (¥)	QALYs	Incremental QALYs	ICER (¥/QALY)
Pre-policy scenario	865,467.53	—	6.75	—	—
Post-policy scenario	2,077,736.48	1,212,268.95	7.26	0.51	2,378,492.79

Abbreviations: ICER, incremental cost-effectiveness ratio; QALY, quality-adjusted life year.

### 3.5 Model validation

The model-predicted cumulative mortality risks at 1, 3, and 5 years were 2.0%, 6.1%, and 10.5%, respectively, under the pre-policy treatment distribution and 1.9%, 5.6%, and 9.4%, respectively, under the post-policy distribution. The corresponding observed mortality risks in the external Chinese registry were 3.1%, 7.1%, and 12.4%. The absolute differences between the pre-policy model predictions and the external estimates were 1.1, 1.0, and 1.9 percentage points at 1, 3, and 5 years, respectively. These findings indicated reasonable agreement between the model predictions and the external cohort, although the model slightly underestimated mortality. Detailed comparisons are provided in Supplementary Table S5.

### 3.6 Sensitivity analysis

**FIGURE 2:**
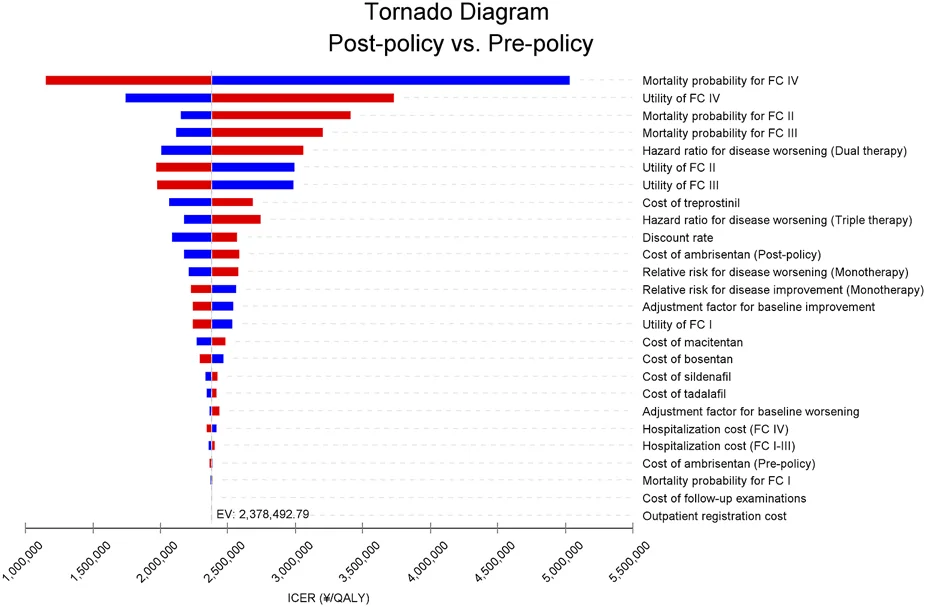
Tornado diagram of one-way sensitivity analyses. The tornado diagram shows the effect on the ICER as each variable varies within its specific range. The vertical solid line represents the value of the ICER in the base-case analysis. The bars represent the ICER ranges obtained by varying each parameter between its lower and upper bounds. Parameters are ordered according to their impact on the ICER. Abbreviations: FC, functional class; ICER, incremental cost-effectiveness ratio; QALY, quality-adjusted life-year.

One-way sensitivity analysis showed that the ICER was most sensitive to the mortality probability and health utility associated with FC IV. Mortality and utility parameters associated with FC II and FC III, as well as the hazard ratio for disease worsening under dual therapy, also had substantial effects on the results. Among the cost parameters, the cost of ambrisentan after policy implementation and the cost of treprostinil had moderate impacts on the ICER. In addition, several state-transition probabilities and the discount rate also contributed to variability in the results. Across all parameter ranges examined, the ICER consistently remained above the WTP threshold, with no threshold crossing observed (Figure 2).

**FIGURE 3:**
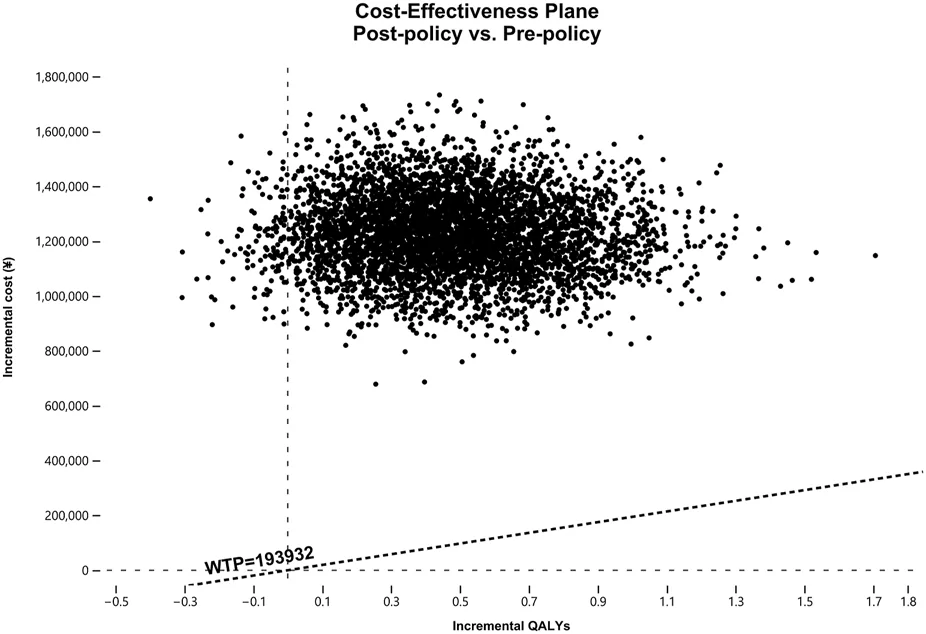
The scatterplot depicts the results of the Monte Carlo analysis. The black dots show 5,000 iterations, and the diagonal dashed line indicates the preset WTP. Abbreviations: WTP, willingness-to-pay. QALY, quality-adjusted life-year.

**FIGURE 4:**
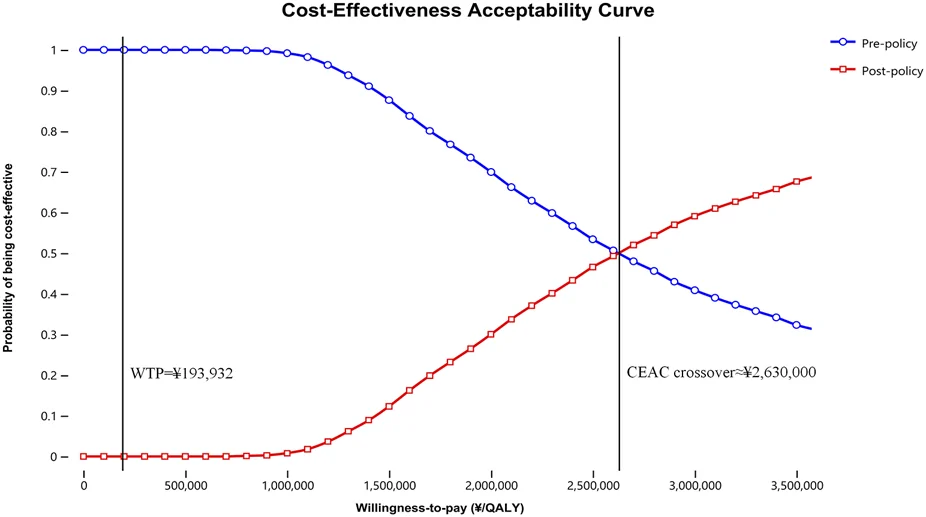
The cost-effectiveness acceptability curve indicates the probability of cost-effectiveness at different WTP thresholds based on the uncertainty of the parameters after 5,000 Monte Carlo simulations. The line consisting of blue circles demonstrates the pre-policy scenario, and the line consisting of red squares shows the post-policy scenario. The vertical solid lines indicate the prespecified WTP threshold of ¥193,932/QALY and the approximate CEAC crossover at ¥2.63 million/QALY. Abbreviations: CNY, Chinese Yuan; QALY, quality-adjusted life year; WTP, willingness-to-pay.

Probabilistic sensitivity analysis further demonstrated that, at a WTP threshold of ¥193,932/QALY, the probability of the post-policy treatment distribution scenario being cost-effective was 0% (Figure 3). The cost-effectiveness acceptability curve (CEAC) showed that the probability of the post-policy treatment distribution scenario becoming the optimal choice exceeded 50% only when the WTP threshold increased to approximately ¥2.63 million/QALY (Figure 4).

### 3.7 Scenario analysis

#### 3.7.1 System-wide price threshold analysis

Scenario analyses initially evaluated the economic impact of price reductions for individual drugs. The results showed that even under the extreme assumption that the costs of treprostinil or the post-policy cost of ambrisentan were reduced to zero, the ICER of the post-policy treatment distribution scenario remained as high as ¥1,140,089/QALY and ¥1,553,550/QALY, respectively, both substantially exceeding the WTP threshold (Table 6).

**TABLE 6:** Results of the scenario analyses.

Scenarios	Incremental cost (¥)	Incremental QALYs	ICER (¥/QALY)
System-wide price threshold analysis			
Post-policy cost of ambrisentan = 0	791,812.53	0.51	1,553,549.97
Cost of treprostinil = 0	581,079.90	0.51	1,140,088.90
Post-policy ambrisentan cost set to 30% of the base-case settlement price	917,949.45	0.51	1,801,032.82
Post-policy ambrisentan cost set to 40% of the base-case settlement price	959,995.09	0.51	1,883,527.10
Targeted therapy prices reduced by 50%	591,946.72	0.51	1,161,409.77
Targeted therapy prices reduced by 70%	343,817.83	0.51	674,576.56
Targeted therapy prices reduced by 80%	219,753.38	0.51	431,159.96
Targeted therapy prices reduced by 90%	95,688.93	0.51	187,743.36
Time horizon scenarios			
5 years	392,984.23	0.08	4,676,291.58
10 years	685,156.44	0.21	3,285,872.28
Disease-stage intervention scenarios			
Early intervention (Initial state: FC II)	1,303,773.62	0.85	1,534,526.44
Late intervention (Initial state: FC III)	1,188,564.88	0.46	2,598,662.69

Abbreviations: FC, functional class; ICER, incremental cost-effectiveness ratio; QALY, quality-adjusted life-year.

In hypothetical scenarios in which the post-policy ambrisentan cost input was reduced to 30% and 40% of its base-case settlement price, the corresponding ICERs were ¥1,801,033/QALY and ¥1,883,527/QALY, respectively (Table 6).

When the prices of all targeted therapies were reduced simultaneously, the ICER progressively decreased as drug prices declined. When drug prices decreased by 50%, 70%, 80%, and 90%, the ICER decreased to ¥1,161,410/QALY, ¥674,577/QALY, ¥431,160/QALY, and ¥187,743/QALY, respectively (Table 6).

**FIGURE 5:**
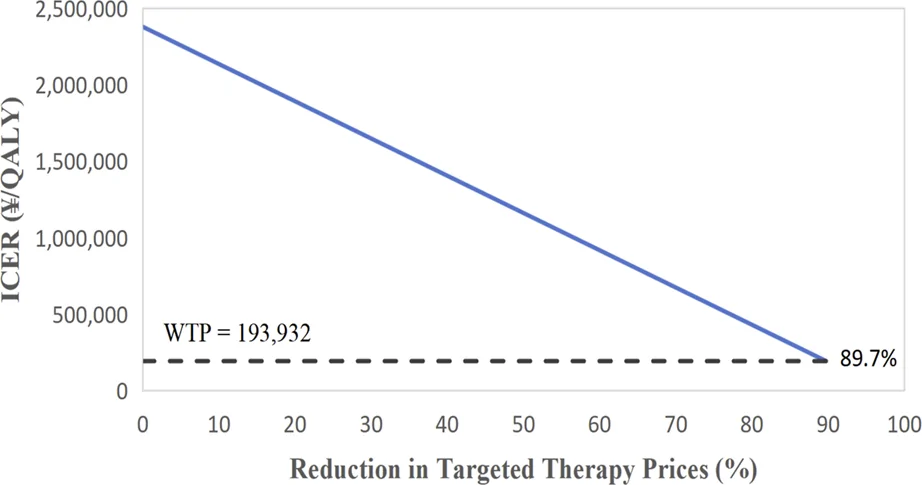
Threshold analysis of targeted therapy price reduction and corresponding ICER changes under the post-policy treatment distribution scenario. The curve illustrates the relationship between reductions in overall targeted therapy prices and the resulting ICER values. The horizontal dashed line represents the predefined WTP threshold of ¥193,932/QALY. The intersection point indicates that the scenario becomes cost-effective only when overall targeted therapy prices are reduced by approximately 89.7%. Abbreviations: ICER, incremental cost-effectiveness ratio; QALY, quality-adjusted life year; WTP, willingness-to-pay.

The results indicated that an overall price reduction of approximately 89.7% would be required for the post-policy treatment distribution to become cost-effective (Figure 5).

#### 3.7.2 Time-horizon scenario analysis

Shortening the model time horizon further increased the ICER, whereas extending the time horizon facilitated the distribution of initial high costs over a longer period and allowed the accumulation of long-term health benefits (Table 6).

#### 3.7.3 Disease-stage intervention analysis

In the early-intervention scenario in which all patients initially entered the model in FC II, the post-policy treatment distribution scenario generated an incremental gain of 0.85 QALYs, corresponding to an ICER of ¥1,534,526/QALY. In contrast, in the late-intervention scenario in which all patients initially entered the model in FC III, the incremental QALY gain was only 0.46, and the ICER increased to ¥2,598,663/QALY (Table 6).

## 4 Discussion

This real-world pharmacoeconomic study showed that implementation of the regional reimbursement policy was associated with increased utilization of combination therapy among patients with PAH. Although the post-policy treatment distribution scenario generated projected long-term health gains in the model, these benefits were accompanied by substantially increased healthcare expenditures, resulting in ICERs far exceeding the conventional WTP threshold.

Unlike traditional pharmacoeconomic studies of PAH that primarily evaluated individual drugs or predefined treatment regimens (Chen et al., 2009; Coyle et al., 2016; Tran-Duy et al., 2021; Buendía et al., 2023; Papademetriou et al., 2023; Dong et al., 2025), the present study focused on reimbursement-policy-associated redistribution of real-world treatment intensity and its projected long-term economic consequences. The model was constructed based on observed treatment distributions before and after policy implementation, thereby characterizing the association between policy implementation and treatment intensity in real-world clinical practice.

One of the principal findings of this study was that implementation of the reimbursement policy was associated with a substantial increase in combination therapy utilization among patients with PAH. Following policy implementation, the proportion of patients receiving combination therapy increased markedly from 27.4% to 80.5%, whereas the proportions receiving no targeted therapy or monotherapy declined substantially. This trend was generally consistent with current guideline recommendations emphasizing earlier intensified therapy in PAH management (Humbert et al., 2022). These findings suggest that reimbursement policy implementation was associated not only with differences in access to targeted therapies but also with broader differences in real-world treatment patterns and treatment intensity.

This study further examined the sequential associations among reimbursement policy implementation, ambrisentan use, and treatment patterns. The association between policy implementation and combination therapy was attenuated, but not eliminated, after ambrisentan use was included in the model. This pattern was descriptively consistent with a possible role for increased ambrisentan use, while other contemporaneous changes may also have contributed. However, because ambrisentan use and treatment regimen were measured during the same index hospitalization, these analyses do not establish a causal or mediating pathway. Accordingly, the findings should be interpreted as exploratory associations rather than evidence that the policy caused treatment escalation. Similar associations between reimbursement changes and drug utilization have been reported in other therapeutic areas (Liu et al., 2023; Zhang et al., 2024). As an important representative of ERAs, reduced ambrisentan costs may have lowered the financial barriers associated with combination therapy and may have been associated with greater uptake of dual and triple combination regimens in clinical practice (Galiè et al., 2015a; Humbert et al., 2022).

Although implementation of the reimbursement policy was associated with substantial treatment intensification, the ICER of the post-policy treatment distribution scenario remained far above the conventional WTP threshold in China, reaching ¥2,378,493/QALY. Probabilistic sensitivity analysis further demonstrated that the probability of the post-policy treatment distribution scenario being cost-effective under the conventional threshold was close to zero. Importantly, the lack of cost-effectiveness of the post-policy treatment distribution should not be interpreted as evidence that the reimbursement policy should be withdrawn. The present analysis compared the observed pre- and post-policy treatment distributions; it neither evaluated a policy-withdrawal scenario nor established that the pre-policy distribution was itself cost-effective. Although the pre-policy distribution was less costly, it also generated fewer projected QALYs and reflected substantially lower utilization of targeted and combination therapies. For a severe and progressive rare disease such as PAH, reimbursement decisions must also consider treatment access, equity, unmet clinical need, and financial risk protection. Therefore, the appropriate policy implication is to maintain treatment access while improving economic efficiency through broader price optimization and more precise risk-stratified treatment, rather than simply reversing reimbursement coverage. Similar economic challenges have also been reported in previous domestic and international studies, suggesting that the long-term pharmacoeconomic performance of PAH combination therapy remains constrained by persistently high medication costs despite reported clinical benefits (Coyle et al., 2016; Buendía et al., 2023; Dong et al., 2023; Papademetriou et al., 2023).

The lack of cost-effectiveness associated with combination therapy observed in this study may be attributable to several structural and methodological factors. First, PAH combination therapy generally requires the long-term or even lifelong concomitant use of two or more high-cost targeted therapies, resulting in continuously accumulating medication costs as treatment intensity increases. In contrast, the associated health benefits often accumulate gradually over a prolonged period, leading to a temporal mismatch between costs and benefits. Second, because previous evidence regarding whether PAH-targeted therapies confer an independent survival benefit remains inconsistent, this study adopted a relatively conservative modeling strategy in which treatments improved prognosis primarily through delaying disease progression rather than directly reducing mortality risk (Macchia et al., 2007; Galiè et al., 2009; Coyle et al., 2016). Furthermore, owing to the lack of high-quality, long-term real-world follow-up data, our model conservatively constrained the cumulative functional improvement potential of intensified combination regimens. While these conservative assumptions were necessary to maintain internal model consistency in the absence of robust longitudinal evidence, they inherently predispose the evaluation toward an underestimation of the long-term QALY gains associated with treatment escalation (Chen et al., 2009; Tran et al., 2015; Coyle et al., 2016).

The scenario analyses further demonstrated that the economic challenges associated with current PAH management are driven primarily by the overall long-term cost structure of multidrug combination therapy rather than by any individual high-cost drug alone. Even when the costs of individual drugs such as ambrisentan or treprostinil were hypothetically reduced to zero, the ICER remained substantially above the WTP threshold. In contrast, only when the prices of all targeted therapies were simultaneously reduced did the overall economic outcomes gradually approach acceptable levels. These findings suggest that reducing the price of a single drug alone may be insufficient to fundamentally improve the long-term cost-effectiveness of combination therapy for PAH. Future improvements in the efficiency of healthcare resource allocation for PAH may therefore require sustained system-wide drug price adjustments together with more precise risk-stratified treatment strategies. As multiple PAH-targeted therapies have been progressively incorporated into the NRDL since 2020 and have subsequently undergone continuous price adjustments, the long-term pharmacoeconomic landscape of PAH treatment may continue to improve in the future (National Healthcare Security Administration, 2020).

In addition, disease-stage-stratified analyses demonstrated that early intervention may yield more favorable economic outcomes than late intervention. In the FC II scenario, patients achieved substantially greater incremental QALY gains and relatively lower ICERs compared with the FC III scenario. These findings further support the current clinical emphasis on early identification and intervention in PAH management (Pulmonary Embolism and Pulmonary Vascular Disease Group of the Chinese Thoracic Society, 2021). Patients at earlier disease stages generally have a longer window of opportunity for clinical benefit and are more likely to maintain stable functional status and quality of life (Dardi et al., 2024), whereas patients at advanced stages experience more rapid disease progression, thereby limiting the extent of reversible health loss even under intensified combination therapy. From a policy perspective, these findings also suggest that improving early detection of PAH and promoting earlier standardized treatment may help improve both clinical outcomes and long-term economic efficiency.

Several limitations of the present study should be acknowledged. First, this was a single-center retrospective study with a relatively limited sample size, and some subgroup analyses included only small numbers of patients. Accordingly, the generalizability of these findings to other institutions or regions should be interpreted with appropriate caution. Although the overall cohort was relatively large for a rare disease, it reflected the accumulation of eligible patients over a 43-month period. As a retrospective before-and-after study spanning several years, the analysis may have been affected by unmeasured secular changes in clinical practice and treatment availability that could not be fully separated from the association with reimbursement policy implementation. In addition, the unequal lengths of the pre-policy and post-policy observation periods should be considered when interpreting the difference in group sizes.

Because sparse-event distributions were present in several exposure categories, certain regression estimates were associated with wide confidence intervals. Therefore, these OR estimates should be interpreted primarily as indicators of association direction and relative magnitude rather than precise effect-size estimates. Although multivariable adjustment was performed, residual confounding remains possible. Potential unmeasured or incompletely measured factors include evolving treatment recommendations and clinical practice, changes in the availability and prices of other PAH-targeted therapies, and physician prescribing preferences. Because the study lacked a concurrent control group, these secular changes could not be separated from reimbursement policy implementation and may have either exaggerated or attenuated the observed associations. These limitations do not alter the descriptive finding that treatment distributions differed markedly between the two periods, but they limit the extent to which these differences can be causally attributed to the reimbursement policy. In addition, because ambrisentan use and treatment regimen were ascertained during the same index hospitalization, the temporal ordering required for a formal mediation analysis could not be established. Furthermore, comparisons of odds ratios across logistic regression models may partly reflect the non-collapsibility of the odds ratio rather than mediation alone. Accordingly, the sequential regression models should be interpreted as exploratory association models rather than as estimates of direct or indirect causal effects.

Second, owing to the lack of long-term Chinese population-specific follow-up data for PAH, some transition probabilities and utility values were derived from international studies. In addition, efficacy parameters for combination therapy were primarily derived from randomized controlled trials, which may not fully reflect treatment adherence, persistence, and heterogeneous clinical characteristics observed in real-world clinical practice. Although parameter uncertainty was evaluated through sensitivity analyses, these extrapolations may still have influenced model outcomes. An external comparison showed that the model-predicted 1-, 3-, and 5-year mortality risks were broadly consistent with those observed in a large Chinese prospective registry, although mortality was slightly underestimated. However, because the external registry consisted specifically of patients with PAH associated with congenital heart disease, differences in etiologic composition may limit direct comparability with the broader PAH population included in the present study.

In addition, relatively conservative methodological assumptions were adopted in the model, particularly regarding mortality benefits and functional improvement, which may have led to conservative estimates of the long-term economic benefits associated with intensified combination therapy (Chen et al., 2009; Tran et al., 2015; Dong et al., 2023). Although these assumptions helped avoid overestimation of long-term treatment effects, they may also have limited the projected long-term QALY gains associated with treatment escalation. Given that PAH remains a chronic progressive disease despite advances in targeted therapies, long-term stabilization rather than persistent reversal of functional status may better reflect routine real-world disease trajectories in many patients. Furthermore, owing to limited availability of longitudinal real-world data, the model did not explicitly account for treatment adherence, adverse-event-related costs, or treatment discontinuation patterns, all of which may influence long-term economic outcomes in real-world clinical practice. Finally, the present analysis was conducted exclusively from the healthcare system perspective and did not incorporate indirect costs, such as productivity loss and caregiver burden; therefore, the broader societal economic burden associated with PAH may not have been fully captured.

In conclusion, implementation of the regional reimbursement policy was associated with substantial changes in real-world treatment patterns among patients with PAH, including a shift toward intensified combination therapy. Although the post-policy treatment distribution generated projected long-term health gains, the resulting treatment distribution scenario remained substantially above the conventional WTP threshold under the prevailing pricing structure. These findings should not be interpreted as support for withdrawing reimbursement coverage. Rather, they indicate that maintaining treatment access while improving economic efficiency may require both targeted price negotiations for individual drugs and broader system-wide price optimization across combination therapy regimens. Earlier risk-stratified intervention may also remain important for improving long-term clinical outcomes and economic efficiency in PAH management.

## Data Availability

The raw data supporting the conclusions of this article will be made available by the authors, without undue reservation.
