# Supplementary material for: Association between a regional reimbursement policy and treatment escalation in pulmonary arterial hypertension: a real-world cohort study with long-term Markov cost-effectiveness analysis
Source: Front Pharmacol. 2026 Aug 27;17:1892453. doi: 10.3389/fphar.2026.1892453 (PMC13558091; doi:10.3389/fphar.2026.1892453)
Supplement: Supplementary file 1 [file Supplementaryfile1.docx]

**CHEERS 2022 Checklist**

| **Section** | **Item** | **No.** | **Guidance for reporting** | **Reported in section** |
| --- | --- | --- | --- | --- |
| **TITLE** | Title | 1 | Identify the study as an economic evaluation and specify the interventions being compared. | Title. The title identifies the study as a long-term Markov cost-effectiveness analysis and identifies the pre-/post-policy treatment context. |
| **ABSTRACT** | Abstract | 2 | Provide a structured summary that highlights context, key methods, results, and alternative analyses. | Abstract. Reports background, objective, design, perspective, Markov approach, costs, QALYs, ICER, sensitivity findings, and conclusion. |
| **INTRODUCTION** | Background and objectives | 3 | Give the context for the study, the study question, and its practical relevance for decision making in policy or practice. | Introduction. Describes the clinical and policy context, evidence gap, study objective, and relevance to reimbursement decision making. |
| **METHODS** | Health economic analysis plan | 4 | Indicate whether a health economic analysis plan was developed and where available. | No separate health economic analysis plan was developed or publicly registered. The methods, assumptions, and analyses of the economic evaluation are described in Sections 2.3–2.9 of the manuscript. |
| **METHODS** | Study population | 5 | Describe characteristics of the study population (such as age range, demographics, socioeconomic, or clinical characteristics). | Methods, Section 2.1; Results, Section 3.1 and Table 2. Describes eligibility, cohort size, study period, baseline clinical characteristics, and treatment distribution. |
| **METHODS** | Setting and location | 6 | Provide relevant contextual information that may influence findings. | Methods, Section 2.1. Reports the Hunan Province policy setting and the single tertiary referral hospital with a pulmonary vascular disease subspecialty. |
| **METHODS** | Comparators | 7 | Describe the interventions or strategies being compared and why chosen. | Methods, Section 2.3; Section 2.5.1. Compares observed pre-policy and post-policy treatment-distribution scenarios across supportive treatment, monotherapy, dual therapy, and triple therapy branches. |
| **METHODS** | Perspective | 8 | State the perspective(s) adopted by the study and why chosen. | Methods, Section 2.3; Section 2.5. Chinese healthcare-system perspective; only direct medical costs were included. |
| **METHODS** | Time horizon | 9 | State the time horizon for the study and why appropriate. | Methods, Section 2.3. Lifetime horizon with 3-month cycles; alternative 5- and 10-year horizons are described in Section 2.8. |
| **METHODS** | Discount rate | 10 | Report the discount rate(s) and reason chosen. | Methods, Table 1; Section 2.6. Costs and health outcomes were discounted at 5% annually; 0% and 8% were examined in sensitivity analyses. |
| **METHODS** | Selection of outcomes | 11 | Describe what outcomes were used as the measure(s) of benefit(s) and harm(s). | Methods, Sections 2.5.4 and 2.6. Outcomes were total costs, QALYs, and ICERs; QALYs captured health benefits across WHO functional-class states. |
| **METHODS** | Measurement of outcomes | 12 | Describe how outcomes used to capture benefit(s) and harm(s) were measured. | Methods, Section 2.4; Section 2.5.4. Disease progression was represented by WHO functional-class transitions; QALYs were calculated from time spent in each state weighted by its utility. |
| **METHODS** | Valuation of outcomes | 13 | Describe the population and methods used to measure and value outcomes. | Methods, Table 1; Section 2.5.4. WHO functional-class utilities were obtained from Keogh et al., based on SF-36 mapping; uncertainty was assessed in sensitivity analyses. |
| **METHODS** | Measurement and valuation of resources and costs | 14 | Describe how costs were valued. | Methods, Table 1; Sections 2.5.1–2.5.3. Reports weighted regimen costs, drug prices, micro-costing for treprostinil, outpatient/examination costs, and FC-specific hospitalization costs. |
| **METHODS** | Currency, price date, and conversion | 15 | Report the dates of the estimated resource quantities and unit costs, plus the currency and year of conversion. | Methods, Section 2.5,; Sections 2.5.2–2.5.3; Section 2.6. Costs are in 2018 CNY; 2018 drug prices required no adjustment, and non-drug costs were deflated to 2018 using the healthcare CPI. |
| **METHODS** | Rationale and description of model | 16 | If modelling is used, describe in detail and why used. Report if the model is publicly available and where it can be accessed. | Methods, Section 2.3 and Figure 1; Section 2.4. Describes the rationale, health states, cycle structure, treatment branches, transitions, and input derivation. The model is not publicly available. |
| **METHODS** | Analytics and assumptions | 17 | Describe any methods for analysing or statistically transforming data, any extrapolation methods, and approaches for validating any model used. | Methods, Section 2.4; Section 2.5.1; Sections 2.7–2.9. Reports transformations of RRs/HRs, mortality conversion, costing equations, structural assumptions, extrapolation, sensitivity analyses, scenarios, and external validation. |
| **METHODS** | Characterizing heterogeneity | 18 | Describe any methods used for estimating how the results of the study vary for subgroups. | Methods, Section 2.8; Results, Section 3.7 and Table 6. Heterogeneity was explored through disease-stage scenarios with all patients entering in FC II versus FC III. |
| **METHODS** | Characterizing distributional effects | 19 | Describe how impacts are distributed across different individuals or adjustments made to reflect priority populations. | Not applicable. No formal distributional cost-effectiveness or equity analysis was undertaken. Equity and access implications are discussed qualitatively in the Discussion. |
| **METHODS** | Characterizing uncertainty | 20 | Describe methods to characterize any sources of uncertainty in the analysis. | Methods, Section 2.7; Table 1; Results, Section 3.6; Section 3.7. Includes one-way, probabilistic, scenario, and threshold analyses. |
| **METHODS** | Approach to engagement with patients and others affected by the study | 21 | Describe any approaches to engage patients or service recipients, the general public, communities, or stakeholders (for example, clinicians or payers) in the design of the study. | No patients, service recipients, members of the public, community representatives, payers, or other external stakeholders were formally involved in the design, conduct, analysis, or reporting of this study. This was a retrospective study based on existing electronic medical record data. |
| **RESULTS** | Study parameters | 22 | Report all analytic inputs (for example, values, ranges, references), including uncertainty or distributional assumptions. | Methods, Table 1; Sections 2.4–2.5; Supplementary Table S4. Reports base-case values, ranges, distributions, sources, and detailed monotherapy weighting inputs. |
| **RESULTS** | Summary of main results | 23 | Report the mean values for the main categories of costs and outcomes of interest and summarize them in the most appropriate overall measure. | Results, Section 3.4 and Table 5. Reports total costs and QALYs for each scenario, incremental costs and QALYs, and the ICER. |
| **RESULTS** | Effect of uncertainty | 24 | Describe how uncertainty about analytic judgments, inputs, or projections affects findings. Report the effect of choice of discount rate and time horizon, if applicable. | Results, Section 3.6; Section 3.7 and Table 6. Reports tornado analysis, probabilistic results, CEAC, price-threshold analysis, alternative time horizons, and disease-stage scenarios. |
| **RESULTS** | Effect of engagement with patients and others affected by the study | 25 | Report on any difference patient or service-recipient, general-public, community, or stakeholder involvement made to the approach or findings of the study. | Not applicable because no patient, public, community, or stakeholder engagement is reported for the study. |
| **DISCUSSION** | Study findings, limitations, generalizability, and current knowledge | 26 | Report key findings, limitations, ethical or equity considerations not captured, and how these could impact patients, policy, or practice. | Discussion and Conclusion. Summarizes findings, relation to prior evidence, policy implications, equity considerations, limitations, generalizability, model assumptions, and remaining uncertainty. |
| **OTHER RELEVANT INFORMATION** | Source of funding | 27 | Describe how the study was funded and any role of the funder in the identification, design, conduct, and reporting of the analysis. | Funding. Reports support from the National Natural Science Foundation of China (Grant No. 82470055). |
| **OTHER RELEVANT INFORMATION** | Conflicts of interest | 28 | Report authors’ conflicts of interest according to journal or International Committee of Medical Journal Editors requirements. | Conflict of Interest. Authors declare no commercial or financial relationships that could be construed as a potential conflict of interest. |
