# Supplementary material for: Association between a regional reimbursement policy and treatment escalation in pulmonary arterial hypertension: a real-world cohort study with long-term Markov cost-effectiveness analysis
Source: Front Pharmacol. 2026 Aug 27;17:1892453. doi: 10.3389/fphar.2026.1892453 (PMC13558091; doi:10.3389/fphar.2026.1892453)
Supplement: Supplementary file 2 [file Table1.docx]

Table S1. Diagnostic criteria for pulmonary arterial hypertension.

| **Domain** | **Diagnostic criterion** |
| --- | --- |
| Hemodynamic confirmation | Mean pulmonary arterial pressure ≥25 mmHg at rest, measured by right heart catheterization |
| Pulmonary arterial wedge pressure | ≤15 mmHg |
| Pulmonary vascular resistance | >3 Wood units |
| Clinical classification | Consistent with World Health Organization Group 1 pulmonary arterial hypertension |
| Exclusion of alternative causes | Exclusion of pulmonary hypertension predominantly caused by left-heart disease, lung disease or hypoxia, chronic thromboembolic disease, or other causes |
| Reference | 2015 ESC/ERS Guidelines for the diagnosis and treatment of pulmonary hypertension |

Table S2. Contingency table analysis of factors associated with ambrisentan use.

| Patient characteristics |  | Number (%) | | χ² | P value |
| --- | --- | --- | --- | --- | --- |
|  |  | Unused | Used |  |  |
| Age, y | ＜26 | 35 (28.9) | 5 (19.2) | 4.455 | 0.216 |
|  | 26-34 | 28 (23.1) | 7 (26.9) |  |  |
|  | 35-46 | 32 (26.4) | 4 (15.4) |  |  |
|  | ＞46 | 26 (21.5) | 10 (38.5) |  |  |
| Gender | Male | 41 (33.9) | 7 (26.9) | 0.472 | 0.492 |
|  | Female | 80 (66.1) | 19 (73.1) |  |  |
| WHO FC | I | 2 (1.7) | 0 (0.0) |  | 0.215 |
|  | Ⅱ | 41 (33.9) | 4 (15.4) |  |  |
|  | Ⅲ | 58 (47.9) | 16 (61.5) |  |  |
|  | Ⅳ | 20 (16.5) | 6 (23.1) |  |  |
| NT-proBNP | ＜300 ng/mL | 7 (5.8) | 1 (3.8) |  | 1.000 |
|  | 300-1100 ng/mL | 22 (18.2) | 4 (15.4) |  |  |
|  | ＞1100 ng/mL | 92 (76.0) | 21 (80.8) |  |  |
| Comorbidities | 0 | 53 (43.8) | 11 (42.3) |  | 0.448 |
|  | 1-2 | 48 (39.7) | 8 (30.8) |  |  |
|  | ≥3 | 20 (16.5) | 7 (26.9) |  |  |
| Location of residence | Urban | 40 (33.1) | 13 (50.0) | 2.664 | 0.103 |
|  | Rural | 81 (66.9) | 13 (50.0) |  |  |
| Occupation | Employed | 21 (17.4) | 8 (30.8) |  | 0.223 |
|  | Retired | 7 (5.8) | 2 (7.7) |  |  |
|  | Unemployed/Farmer | 93 (76.9) | 16 (61.5) |  |  |
| Type of insurance system | Urban employee | 10 (8.3) | 5 (19.2) |  | <0.001 |
|  | Urban-rural resident | 35 (28.9) | 17 (65.4) |  |  |
|  | Out-of-pocket | 76 (62.8) | 4 (15.4) |  |  |
| Implementation of the policy | Before | 104 (86.0) | 2 (7.7) | 65.170 | <0.001 |
|  | After | 17 (14.0) | 24 (92.3) |  |  |

Abbreviations: WHO FC, World Health Organization Functional Class; NT-proBNP, N-terminal pro-brain natriuretic peptide.

Table S3. Contingency table analysis of factors associated with targeted treatment regimens.

| Patient characteristics |  | Number (%) | | | | χ² | P value | |
| --- | --- | --- | --- | --- | --- | --- | --- | --- |
|  |  | No targeted therapy | Monotherapy | Dual therapy | Triple therapy |  | |  |
| Age, y | ＜26 | 6 (15.8) | 21 (44.7) | 10 (19.2) | 3 (30.0) |  | | 0.044 |
|  | 26-34 | 9 (23.7) | 7 (14.9) | 16 (30.8) | 3 (30.0) |  | |  |
|  | 35-46 | 11 (28.9) | 10 (21.3) | 15 (28.8) | 0 (0.0) |  | |  |
|  | ＞46 | 12 (31.6) | 9 (19.1) | 11 (21.2) | 4 (40.0) |  | |  |
| Gender | Male | 12 (31.6) | 16 (34.0) | 16 (30.8) | 4 (40.0) |  | | 0.942 |
|  | Female | 26 (68.4) | 31 (66.0) | 36 (69.2) | 6 (60.0) |  | |  |
| WHO FC | I | 2 (5.3) | 0 (0.0) | 0 (0.0) | 0 (0.0) |  | | <0.001 |
|  | Ⅱ | 16 (42.1) | 19 (40.4) | 10 (19.2) | 0 (0.0) |  | |  |
|  | Ⅲ | 14 (36.8) | 21 (44.7) | 35 (67.3) | 4 (40.0) |  | |  |
|  | Ⅳ | 6 (15.8) | 7 (13.5) | 7 (13.5) | 6 (60.0) |  | |  |
| NT-proBNP | ＜300 ng/mL | 2 (5.3) | 5 (10.6) | 1 (1.9) | 0 (0.0) |  | | 0.324 |
|  | 300-1100 ng/mL | 9 (23.7) | 7 (14.9) | 10 (19.2) | 0 (0.0) |  | |  |
|  | ＞1100 ng/mL | 27 (71.1) | 35 (74.5) | 41 (78.8) | 10 (100.0) |  | |  |
| Comorbidities | 0 | 13 (34.2) | 23 (48.9) | 25 (48.1) | 3 (30.0) |  | | 0.067 |
|  | 1-2 | 22 (57.9) | 14 (29.8) | 17 (32.7) | 3 (30.0) |  | |  |
|  | ≥3 | 3 (7.9) | 10 (21.3) | 10 (19.2) | 4 (40.0) |  | |  |
| Location of residence | Urban | 11 (28.9) | 16 (34.0) | 22 (42.3) | 4 (40.0) |  | | 0.592 |
|  | Rural | 27 (71.1) | 31 (66.0) | 30 (57.7) | 6 (60.0) |  | |  |
| Occupation | Employed | 7 (18.4) | 6 (12.8) | 16 (30.8) | 0 (0.0) |  | | 0.138 |
|  | Retired | 2 (5.3) | 2 (4.3) | 4 (7.7) | 1 (10.0) |  | |  |
|  | Unemployed/Farmer | 29 (76.3) | 39 (83.0) | 32 (61.5) | 9 (90.0) |  | |  |
| Type of insurance system | Urban employee | 3 (7.9) | 1 (2.1) | 11 (21.2) | 0 (0.0) |  | | <0.001 |
|  | Urban-rural resident | 8(21.1) | 13 (27.7) | 23 (44.2) | 8 (80.0) |  | |  |
|  | Out-of-pocket | 27 (71.1) | 33 (70.2) | 18 (34.6) | 2 (20.0) |  | |  |
| Implementation of the policy | Before | 37 (97.4) | 40 (85.1) | 28 (53.8) | 1 (10.0) |  | | <0.001 |
|  | After | 1 (2.6) | 7 (14.9) | 24 (46.2) | 9 (90.0) |  | |  |
| Ambrisentan use | Unused | 38 (100.0) | 46 (97.9) | 34 (65.4) | 3 (30.0) |  | | <0.001 |
|  | Used | 0 (0.0) | 1 (2.1) | 18 (34.6) | 7 (70.0) |  | |  |

Table S4. Drug-utilization proportions and efficacy parameters used to derive the composite relative risks for monotherapy.

| **Monotherapy** | **Number, n** | **Utilization proportion** | **RR for WHO FC improvement**  **(95% CrI)** | **RR for WHO FC worsening**  **(95% CrI)** | **Source** |
| --- | --- | --- | --- | --- | --- |
| Bosentan | 26 | 55.32% | 2.05 (1.25–3.32) | 0.46 (0.18–1.04) | Coyle et al. (2016). |
| Ambrisentan  10 mg | 1 | 2.13% | 1.21 (0.62–2.23) | 0.25 (0.05–0.81) | Coyle et al. (2016). |
| Sildenafil | 17 | 36.17% | 3.71 (1.76–7.29) | 0.27 (0.04–1.10) | Coyle et al. (2016). |
| Tadalafil | 2 | 4.26% | 2.67 (1.11–5.76) | 0.45 (0.11–1.44) | Coyle et al. (2016). |
| Subcutaneous treprostinil* | 1 | 2.13% | 9.42 (5.65–17.48) | 0.40 (0.15–0.93) | Epoprostenol proxy from Coyle et al. (2016) |
| **Total/composite estimate** | **47** | **100.00%** | **2.8157** | **0.3851** | Weighted calculation |

CrI, credible interval; FC, functional class; RR, relative risk.

*A corresponding short-term RR for subcutaneous treprostinil was unavailable. The published epoprostenol estimate was therefore used as a prostacyclin-class proxy.

The composite RR for outcome *k* was calculated as:

$${\overline{\text{RR}}}_{\text{k}}\text{=}\sum_{\text{i}\text{=1}}^{\text{m}} \text{w}_{\text{i}}\text{R}\text{R}_{\text{i}\text{,}\text{k}}\text{=}\frac{\sum_{\text{i}\text{=1}}^{\text{m}} \text{n}_{\text{i}}\text{R}\text{R}_{\text{i}\text{,}\text{k}}}{\sum_{\text{i}\text{=1}}^{\text{m}} \text{n}_{\text{i}}}\text{,}$$

Where *k* denoted WHO FC improvement or worsening, *n_i_* was the number of patients receiving drug *i*, *w_i_* was the corresponding utilization proportion, and *RR_i,k_* was the drug-specific RR for WHO FC improvement or worsening reported in the source study.

The numerical calculations were:

$$\text{R}\text{R}_{\text{improvement}}\text{=}\frac{\text{26×2.05+1×1.21+17×3.71+2×2.67+1×9.42}}{\text{47}}\text{=2.8157,}$$

$$\text{R}\text{R}_{\text{worsening}}\text{=}\frac{\text{26×0.46+1×0.25+17×0.27+2×0.45+1×0.40}}{\text{47}}\text{=0.3851.}$$

Table S5. External comparison of model-predicted and observed cumulative mortality risks.

| **Survival outcome** | **Pre-policy model** | **Post-policy model** | **External Chinese registry** |
| --- | --- | --- | --- |
| 1-year mortality | 2.0% | 1.9% | 3.1% |
| 3-year mortality | 6.1% | 5.6% | 7.1% |
| 5-year mortality | 10.5% | 9.4% | 12.4% |

Note: Observed mortality risks were calculated as one minus the reported survival probability. The external registry included patients with pulmonary arterial hypertension associated with congenital heart disease; therefore, the comparison was regarded as supportive external validation.
